# Asthma and Multi-Food Allergy Are Risk Factors for Oral Food Challenge Failure—A Single-Center Experience

**DOI:** 10.3390/nu17172769

**Published:** 2025-08-27

**Authors:** Liliana Klim, Maria Michalik, Ewa Cichocka-Jarosz, Urszula Jedynak-Wąsowicz

**Affiliations:** 1 SSG at Children’s Diseases Clinic, Children’s University Hospital, Jagiellonian University Medical College, 31-008 Krakow, Poland; liliana.klim@student.uj.edu.pl (L.K.); maria02.michalik@student.uj.edu.pl (M.M.); 2 Department of Pediatrics, Children’s University Hospital, Jagiellonian University Medical College, 31-008 Krakow, Poland; ewa.cichocka-jarosz@uj.edu.pl; 3 Department of Pulmonology, Allergy and Dermatology, Children’s University Hospital, 30-663 Krakow, Poland

**Keywords:** food allergy, oral food challenge, anaphylaxis, risk factors, children

## Abstract

**Background:** Diagnosing food allergy (FA) typically involves a detailed clinical history and confirmation of allergen-specific IgE. Oral food challenges (OFCs) remain the gold standard in FA diagnosis. This study aimed to present our experience in performing OFCs in pediatric patients with particular focus on challenges performed with cow’s milk and hen’s egg. **Methods:** We conducted a retrospective analysis of 205 OFCs. Clinical data were evaluated and multiple logistic regression was used to identify associations between challenge outcomes, reaction severity, and comorbidities. **Results:** The mean age of patients was 5.7 ± 3.1 years, with 135 (65.9%) being male. The tested foods included cow’s milk protein (CMP, 103 challenges; 50.2%), hen’s egg white protein (HEWP, 84; 41.0%), peanuts (3; 1.5%), tree nuts (4; 2.0%), gluten (3; 1.5%), hen’s egg yolk (4; 2.0%), and other foods (4; 2.0%). The overall OFC failure rate was 32.2%, and five challenges (2.4%) yielded inconclusive results. The median cumulative reactive dose was 0.27 g for baked CMP and 0.58 g for baked HEWP. Most failed OFCs involved mucocutaneous symptoms (44 cases; 66.7%). Severe multisystemic reactions occurred in four patients (2.0%), all of whom required epinephrine (6.1% of positive challenges). An increased risk of OFC failure was associated with asthma (*p* = 0.028; 95% CI: 0.07–1.27) and multi-food allergy (*p* = 0.021; 95% CI: 0.14–1.67). Additionally, the coexistence of asthma and a prior history of anaphylaxis to any food was related to OFC failure (*p* = 0.049; 95% CI: 0.01–2.19), as was the combination of multi-food allergy and previous anaphylaxis (*p* = 0.043; 95% CI: 0.03–1.70). Receiver operating characteristic (ROC) curve analysis was utilized to predict outcomes of OFCs to baked milk and baked egg and determined a specific IgE (sIgE) cutoff level of 58.1 kU/L for baked milk challenges (AUC: 0.77; sensitivity: 0.588; specificity: 0.882), and 11.3 kU/L for baked egg challenges (AUC: 0.66; sensitivity: 0.692; specificity: 0.607). **Conclusions:** Our findings confirm that OFCs are a safe and effective tool for diagnosing FA in children. With appropriate patient selection, the risk of severe reactions remains low. Nonetheless, comorbidities such as asthma and multi-food allergy are associated with an increased likelihood of OFC failure.

## 1. Introduction

A food allergy (FA) is defined as an adverse reaction to food mediated by an immunologic mechanism, involving specific IgE (IgE-dependent), cell-mediated mechanisms (non-IgE-dependent), or both (mixed IgE- and non-IgE-dependent) [1]. Any food can potentially trigger an allergic reaction [2], with a wide variety of clinical manifestations. The prevalence of FAs has been increasing during the last 2–3 decades in certain regions of the world, which has made it a pivotal topic in medical studies [3]. Food allergies are more likely to affect children rather than adults and appear to disproportionately affect patients in urban/industrialized regions [4,5]. Food allergies are a growing health concern in both middle- and high-income countries; what is more, allergic multimorbidity is often observed [6]. The quality of life of children with FAs and their families is significantly affected by the disease, which has economic, social, and nutritional effects. Discrepancies can be observed among different studies, encompassing varying populations. The occurrence of FAs in European children was estimated between 1.4% and 5.6% [7]. According to a recent systematic review and meta-analysis, the pooled prevalence of self-reported food allergy in European children is approximately 13.1%, while 16.6% show sensitization based on specific IgE testing, and 5.7% have positive skin prick tests. However, the prevalence of clinically confirmed food allergy, verified through oral food challenges (OFCs), is considerably lower and estimated at around 0.8%. When examining individual allergens, the lifetime prevalence based on self-report ranges from 0.5% to 5.7%, with cow’s milk and hen’s egg being the most frequently reported. These findings highlight the discrepancy between perceived and objectively confirmed food allergy and emphasize the importance of accurate diagnostic tools in pediatric populations [8]. One study revealed that specific IgE (sIgE) sensitization in Polish children was most common to peanuts, hazelnuts, and apples [9]. Birch pollen-related FAs were found to explain the high prevalence of hazelnut, apple, peach, kiwi, and carrot FAs in countries such as Switzerland, the Netherlands, Poland, Lithuania, and Scandinavian countries where birch pollen is a common airborne allergen [8,10].

Food allergies require proper management, which includes correct diagnosis and treatment. Establishing a diagnosis of food allergy usually involves a careful clinical history, evidence of allergen-specific IgE by skin and/or blood testing, determination of the total count of eosinophils, basophil activation test, or mast cell activation test [11]. In recent years, component-resolved diagnostics (CRD) have become a recognized and widely used method in the evaluation of IgE-mediated food allergy. Unlike conventional extract-based testing, CRD identifies sensitization to specific allergenic molecules, allowing for a more refined risk assessment. This approach can help in guiding clinical decisions, particularly when evaluating the necessity and safety of oral food challenges (OFCs). However, the latest evidence supports the continued diagnostic value of extract-based IgE testing. A 2024 systematic review and meta-analysis by Riggioni et al. demonstrated that extract-based assays still provide strong diagnostic performance in identifying food allergies [12]. Therefore, CRD should be considered a complementary rather than replacement tool, enhancing but not substituting traditional IgE testing in clinical allergy assessment.

Oral food challenges (OFCs) remain the gold standard for the diagnosis of food allergy, but these procedures are also performed in order to evaluate the effect of food processing on food tolerability or to determine the quantity for safe intake [13]. Moreover, they provide an occasion for food reintroduction [14]. Double-blind, placebo-controlled food challenges (DBPCFCs) are considered to be the criteria standard. Nonetheless, because of methodological difficulties they are rarely conducted in clinical practice, especially in pediatric patients. Open (non-blinded) challenges are therefore the method of choice [15,16]. The accuracy of interpreting serum and skin testing for food allergies has been a prevailing issue in the field of allergology and immunology. The usage of these methods may lead to the mislabeling of the food allergy diagnosis. The discrepancy is due to possible aberrations in user interpretation. Taking these aspects into account, it remains clear that an OFC is the most predictive and accurate method of confirming a food allergy diagnosis [17]. Every food challenge should be standardized and include a physical examination, the monitoring of vital signs before, during, and after the challenge, the administration of food in a planned manner in order to gradually reach the cumulative dose, and finally, the treatment of symptoms if needed [15]. Risk factors for severe reaction during an OFC include a history of anaphylaxis to the causative food, increasing age of the child [18], presence of asthma or atopic dermatitis, performing OFCs to high-risk allergens (peanuts and cashew), physical exercise, and severe infections [13,19,20]. The aim of our study was to share experiences with conducting OFCs in pediatric patients. We sought to evaluate the outcomes and predictors of severe symptoms in OFCs performed in our single-center population. As milk and egg allergies are the most frequently observed in the pediatric population, we intended to consider OFCs involving these two allergens separately and examine them with particular focus.

## 2. Materials and Methods

### 2.1. Study Population and Data Collection

A retrospective data chart review was performed on 205 standard-of-care OFCs conducted between 1 January 2014 and 30 September 2024 in the Pediatric Allergy Department. For this study, we used cases admitted to inpatient care with the aim of conducting an OFC to a specific food allergen. Patients were either suspected or diagnosed with IgE-dependent food allergies. Clinical data, including patients’ gender, age, history of food-induced anaphylaxis, allergic comorbidities (atopic dermatitis, asthma, and multi-food allergy), and family history of atopy were gathered from the databases of the department.

Ethics committee approval was obtained for this study (118.0043.1.274.2025). The requirement for informed consent was waived due to the retrospective nature of the study. An informed consent for the OFC was obtained before every procedure.

### 2.2. Laboratory Tests

A specific IgE antibody detection test was performed as a part of qualifying a patient for an OFC. The specific IgE antibody levels to food allergen extract were measured using the ImmunoCAP^®^ Specific IgE test (Pharmacia Diagnostics, Uppsala, Sweden). The results were obtained within 6 months before the OFC. Results of sIgE > 0.35 kU/L were considered positive.

### 2.3. Oral Food Challenges (OFCs)

Open OFCs were conducted in accordance with the AAAAI–EAACI PRACTALL Guidelines [21,22]. All children who fulfilled eligibility criteria (were in good health on the day of the challenge, with comorbid allergic diseases under optimal control and medications potentially interfering with the procedure (antihistamines, leukotriene antagonists, and systemic steroids) discontinued according to recommendations) [23]. In children with concomitant pollinosis, challenges were not scheduled during the relevant pollen season. For safety reasons, all challenges were performed in a hospital day-care unit, with immediate access to emergency equipment and medications (adrenaline, oxygen, and intravenous fluids). A physician experienced in pediatric anaphylaxis was present throughout the procedure. Vital signs (heart rate, blood pressure, and oxygen saturation) and clinical status were assessed before each incremental dose.

Dosing procedure. Food allergens were administered in incremental doses at 30 min intervals until the planned cumulative dose was achieved.

For the baked milk challenge, the total dose was equal to 32 mL of cow’s milk baked into 2 muffins (doses = 1/4, 1/4, 1/2, and 1 muffin). For the baked egg challenge, the total dose was equal to 1/3 of a large egg (2 eggs per 12 muffins; 2 muffins per challenge; doses = 1/8, 1/8, 1/4, 1/2, and 1 muffin). Other challenges: plain or heated food was used, starting from a minimal dose (≈3 mg protein or 0.1 mL liquid) and progressing to an age-appropriate portion (e.g., 200 mL milk, 1 whole egg, or 3 g of peanut protein).

Observation and stopping criteria. Children were observed continuously during dosing and for at least 4 h after the final dose. Challenges were discontinued if objective allergic symptoms occurred, or if persistent subjective complaints (e.g., pruritus, abdominal pain, nausea, or behavioral change) raised concern for progression [23,24]. If a child refused the food, the OFC outcome was classified as inconclusive, and a continuation of the elimination diet was recommended.

The outcome of the challenge was classified as negative (passed) when the full cumulative dose was consumed without symptoms or as positive (failed) when symptoms of allergic reaction occurred. The challenge team assessed severity according to H.A. Sampson’s five-grade classification for food-induced anaphylaxis [25].

The decision to perform an OFC with baked or raw forms of the allergen was based on the patient’s clinical history, previous allergic reaction to the tested food, their severity, results of allergic tests, and the study objectives. In children with a history of immediate moderate or severe allergic reactions to milk or egg and high levels of specific IgE, challenges were initially performed with extensively heated (baked) forms to assess the acquisition of partial tolerance, in accordance with safety protocols. If tolerance to baked forms was demonstrated in the past, subsequent challenges were considered with less processed or raw milk or egg to evaluate the possibility of complete tolerance. In other cases, when the risk of severe reaction was considered low or when full tolerance was being assessed, OFCs were performed directly with raw food allergens.

### 2.4. Statistical Method

Data were managed using Pandas-Python Data Analysis Library (vs. 3.12). Statistical analysis was performed using Statsoft STATISTICA 13.3., licensed under Jagiellonian University. Descriptive statistics were presented as means and medians for quantitative data and as counts and percentages for qualitative data. The Pearson chi-square test for categorical values together with the Mann–Whitney U test for ordinal and continuous variables were used for inter-group analysis. Spearman’s rank correlation was utilized to establish a correlation between the patient’s age and OFC outcome. A logistic regression model was created using the Python language scikit-learn library and applied to explore associations between comorbidities, clinical history, and the result of an OFC, as well as for the determination of the ROC curves. The sensitivity and specificity were based on the Youden method. Youden’s index (J) is a summary measure of the ROC curve, defined as sensitivity + specificity—1. It provides an optimal balance between sensitivity and specificity and was used in our study to select the most appropriate cutoff values for sIgE. Statistical significance was set to a *p* value less than 0.05.

## 3. Results

### 3.1. General Information

In total, 205 patients were included in this study. Five patients (2.4%) refused to consume the allergen during the procedure (three of them were challenged with cow’s milk and two with hen’s egg). All of them were less than 3 years of age. In these cases, the OFC was regarded as inconclusive. The children with inconclusive OFC outcomes were excluded from the analysis. Finally, complete data were obtained from 200 challenged patients and then evaluated.

Population characteristics, including age, gender, mean time from consumption to symptom onset, allergens challenged, as well as concomitant diseases and family history of atopy are presented in Table 1. A male predominance was observed in the examined group of patients. The most frequently challenged allergens were cow’s milk protein (CMP), amounting to 50.2% (103 cases) of all OFCs conducted, and hen’s egg white protein (HEWP). The majority of patients presented with a multi-food allergy, which was the most prevalent accompanying atopic disease in our cohort. As the vast majority of patients were challenged either with CMP or HEWP, we performed a comparative analysis of these two populations, as presented in Table 2. The only finding was a higher frequency of atopic dermatitis in children sensitized to HEWP.

The overall failure rate for all OFCs was 32.2%, as 66 OFCs were classified as positive (failed). A total of 41 oral food challenges (OFCs) were performed in children under 3 years of age, representing 20.0% of all cases, with six patients (14.6%) failing the test. The majority of OFCs were conducted in children aged 3 to 6 years, comprising 96 cases (46.8%). This age group demonstrated the highest failure rate, with 37 patients (38.5%) not passing the OFC. In children over 6 years of age, 68 OFCs were performed (33.2%), of which 23 (33.8%) resulted in a failed outcome.

Outcomes of all OFCs to different allergenic foods including their preparation form and specific failure rates are presented in Table 3. Among all allergens tested, the highest failure rate was observed in the HEWP group, closely followed by the CMP group.

As the majority of OFCs were performed with CMP and HEWP, we focused on showcasing detailed results of challenges with these two food allergens in Table 4. Other allergen groups were excluded from detailed analysis due to a small sample size and no statistical power. A statistically significant difference was observed in mean cumulative reactive dose values for HEWP and CMP in the baked allergen group (*p* = 0.01). However, there was no statistical difference between cumulative reactive doses for CMP and HEWP in the non-baked allergen group (*p* = 0.06).

### 3.2. Symptoms Reported During Failed OFCs and Treatment

**Figure 1:**
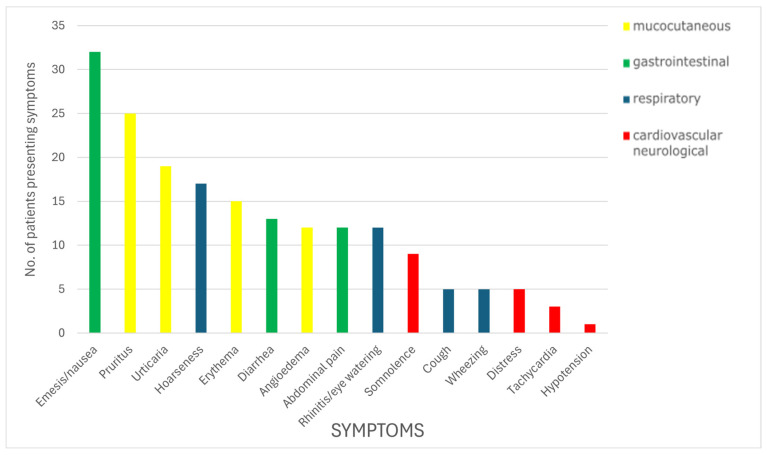
Symptom distribution across different systems with numbers of patients presenting each symptom during failed OFCs.

In this study, most patients who failed an OFC presented with mucocutaneous symptoms (N = 44, 66.7%), followed by gastrointestinal (N = 37, 56.1%) and respiratory symptoms (N = 30, 45.5%). Cardiovascular and neurological manifestations were least common and were observed in 15 patients (22.7%). The symptom distribution across different systems with numbers of patients presenting each symptom is detailed in Figure 1. Distress was defined as overall anxiety or change in behavior that appeared during the procedure, while somnolence referred to sleepiness or drowsiness reported by the patient or the guardian.

Clinical reactions for failed OFCs were graded based on H.A. Sampson’s severity score for food-induced anaphylaxis [25]. Most patients were classified as having grade 2 anaphylaxis, amounting to 31 cases (47.0%). Grade 1 reactions were also commonly recorded in 29 procedures (43.9%). Signs of grade 3 anaphylaxis were observed in two patients (3.0%). It is worth mentioning that in only four procedures (less than 2% of all OFCs performed) patients experienced severe multisystemic reactions—three patients (4.5%)—two challenged with cow’s milk and one with hen’s egg presented with grade 4 symptoms and one patient (1.5%) was diagnosed with grade 5 anaphylactic shock (challenged with cow’s milk). The following day, this one particular patient developed an acute respiratory tract infection of *Mycoplasma pneumoniae* etiology.

The most frequently used medication type during the failed OFCs were antihistamines (46 cases, 69.7%), with 10 patients (15.2%) being administered first-generation antihistamines (clemastine) intravenously and 36 patients (54.5%) being given second-generation drugs from that group (cetirizine or rupatadine) orally. Glucocorticosteroids were administered to 20 patients (30.3%) and fluid therapy was needed in eight cases (12.1%). Epinephrine administration was required in four failed challenges (6.1%)—patients with grade 4 and 5 reactions. The patient with the grade 5 reaction (1.5%) required two doses of intramuscular epinephrine 10 min apart and additionally an epinephrine nebulization due to severe Quincke angioedema and stridor occurrence. The other two patients who needed epinephrine administration were also challenged with baked milk and they presented severe bronchospasm with tachycardia and generalized urticaria. Both had asthma (well controlled at the moment of OFC). The fourth patient who required epinephrine—the girl with Netherton syndrome—developed urticaria and throat angioedema with tachycardia and intractable vomiting after the ingestion of cottage cheese. All of those children were of early school age.

Topical nasal alfa-mimetics (oxymetazoline and ephedrine) were administered to eight patients (12.1%). Short-acting beta-agonists (SABAs) were given in three cases (4.5%) and a combination of a SABA and ipratropium bromide was utilized in two cases (3.0%). In 17 failed OFCs (25.8%), the symptoms resolved spontaneously without any treatment.

### 3.3. Factors Influencing OFC Outcome

The influence of various factors on the OFC outcome based on logistic regression analysis is outlined in Table 5. The results show that the presence of asthma was significantly associated with the possibility of an OFC failure. Another detected risk factor for an OFC failure was multi-food allergy. Further analysis of the correlation between the patient’s age and challenge result did not show a statistically significant association between those two variables (r = −0.03, *p* = 0.66) either. The difference between the prevalence of a family history of atopy in passed and failed OFC groups of patients was not statistically significant. Moreover, we found that a coexistence of asthma and previous history of anaphylaxis to any food was a risk factor for OFC failure. A similar correlation was observed among children with multi-food allergy and a history of anaphylaxis to any food and OFC failure.

In our study, we also attempted to stratify and predict the outcomes of oral food challenges based on the child’s age, dividing the cohort into three age groups: 1–3 years, 3–6 years, and above 6 years. However, we did not find any significant correlations between age and the outcomes of OFC.

Specific IgE (sIgE) values were obtained in 78 patients before performing OFCs with CMP and in 60 patients that underwent the procedure with HEWP. In some children, sIgE values were not obtained, as they were qualified for an OFC based on a skin prick test.

The values of CMP- and HEWP-specific IgE in baked and non-baked allergen groups with a comparison of values in passed and failed OFCs for both triggers are presented in Table 6. There was a statistically significant difference between HEWP-specific IgE median levels in passed and failed OFCs in the baked allergen group; however, such a difference was not discovered for non-baked HEWP. No notable statistical difference was observed in CMP-specific IgE values between passed and failed challenges in both baked and non-baked groups.

A comparison of median values of specific IgE levels for CMP and HEWP in failed baked and non-baked challenges is showcased in Table 7. A statistically significant difference between CMP- and HEWP-specific IgE levels was observed in the baked allergen group with higher levels of CMP-specific IgE than HEWP-specific IgE levels. Such a difference was not present in the non-baked group.

**Figure 2:**
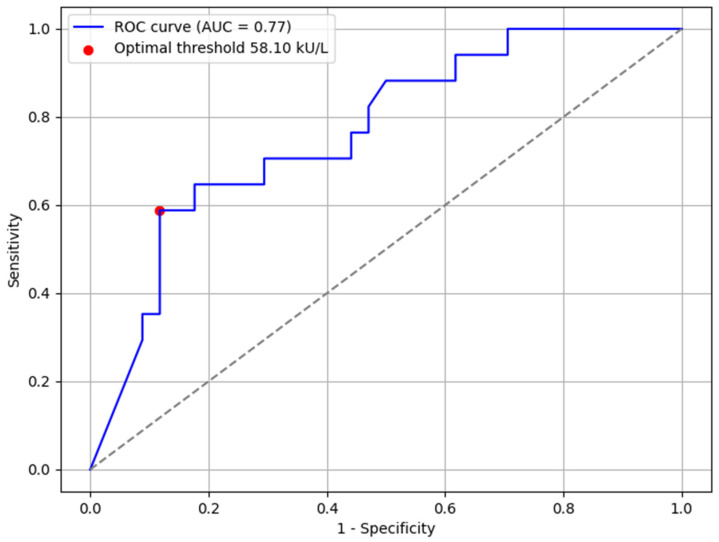
Receiver operating characteristic (ROC) curve for cow’s milk IgE levels. AUC—area under the curve.

**Figure 3:**
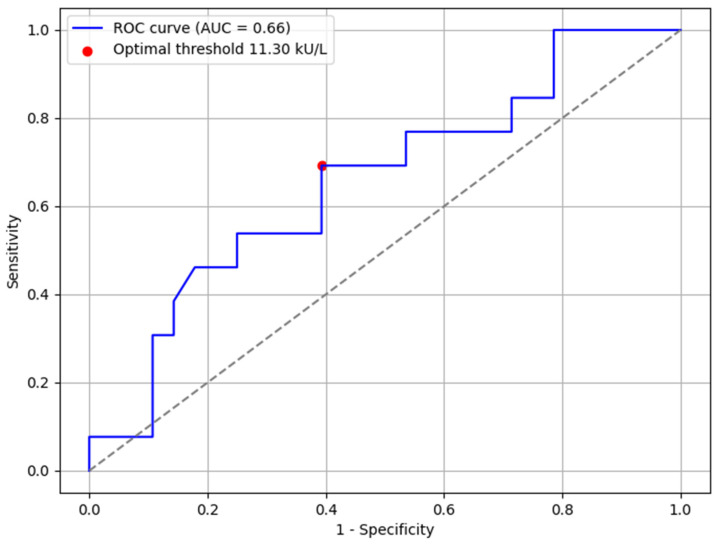
Receiver operating characteristic (ROC) curve for hen’s egg white IgE levels. AUC—area under the curve.

Receiver operating characteristic (ROC) curves were created and analyzed to discover the most optimal cutoff sIgE levels to predict outcomes of OFCs to baked milk and baked egg white. There were not enough data to create ROC curves for other allergen groups. Optimal cutoff points were established in a way that ensured both the safety of an OFC (the highest specificity possible) and had an acceptable Youden’s index. A cutoff sIgE level of 58.1 kU/L (AUC 0.77, sensitivity 0.588, and specificity 0.882) was set for baked milk challenges (Figure 2) and a threshold of 11.3 kU/L (AUC 0.66, sensitivity 0.692, and specificity 0.607) for baked egg challenges (Figure 3). An AUC of 0.77 (for baked milk) reflects an acceptable, moderate discriminatory power. Conversely, an AUC of 0.66 (for baked egg) represents a limited predictive value and should not be considered sufficient for clinical decision-making on its own.

## 4. Discussion

Food allergies remain a common issue in the pediatric population; they contribute to a reduced quality of life in patients and their families [4], as strict avoidance measures must be taken to decrease the risk of food-induced anaphylaxis [26]. This leads to an elimination diet, which can cause nutritional deficiencies [27], and increased anxiety associated with accidental allergen exposure [28,29]. An oral food challenge remains a primary tool that can alleviate these effects and reduce unnecessary food avoidance after a successful completion of the procedure [30,31]. However, many patients and parents are reluctant to consent to OFCs due to fear surrounding the procedure and the risk of an adverse reaction [32]. Therefore, we performed a meticulous analysis of OFCs and clinical criteria, which could be utilized to predict the outcome of the procedure and improve its safety.

As observed in other studies, our research found a predominance of male patients [33,34,35,36,37], with 135 (over 65%) procedures being performed in boys. Males frequently exhibit a stronger type 2 immune response, which induces more prevalent allergic sensitization and therefore increased demand for OFCs in this group of patients. In contrast, females tend to develop a more balanced type 1/type 2 immune response, which may determine the observed gender differences in allergy prevalence [38]. Moreover, studies have shown that boys are inclined to have higher total and allergen-specific IgE levels in comparison with girls, which are associated with a higher prevalence of food allergies [39] and food-induced anaphylaxis in males [40,41]. The occurrence of more severe reactions and higher failure rates among male patients undergoing OFCs was also observed in some studies [42,43].

Identifying patients who would be ideal candidates for OFCs is a demanding process, since there are multiple factors that potentially influence the outcome of the procedure. Our retrospective analysis of 205 OFCs showed an overall failure rate of 32.2% (N = 66), which is comparable to results of previous studies, ranging from 8.7% to 85% [20,33,35,44,45]. Acceptable failure rates depend on the type of center where the procedures are performed, and may be higher in clinics with access to advanced care and the possibility of treating patients experiencing severe symptoms and where high risk OFCs are carried out, whereas low positivity rates are desired in outpatient settings because of limited possibilities for enhanced treatment and these are where the patients with a low risk of OFC failure are qualified [44].

Among all allergens tested in our patient population, the highest failure rate was observed in the HEWP group (N = 29, 34.5%), closely followed by CMP with 32 recorded failures (31.1%). In line with other studies, the most commonly challenged allergens in our cohort were CMP and HEWP [37,46]. According to research findings, these two allergens are also the most frequent causes of anaphylactic reactions [47]. Other allergenic foods tested, including peanuts and tree nuts, were not as prevalent in our study as in other populations [20,36]. This discrepancy reflects regional dietary patterns; in Poland, children, especially infants, are less likely to consume products based on nuts than in some Western countries. Therefore, early screening and food allergy testing mainly includes allergens such as CMP and HEWP, as these foods are widely consumed in this age group.

Our study found that mucocutaneous symptoms were most frequent among patients who failed an OFC, aligning with other research findings [33,35,44,45,48,49,50,51]. Gastrointestinal manifestations were more prevalent in our cohort than respiratory symptoms; a similar trend was observed by Ballini et al. [33] and Yanagida et al. [52]. Some authors, however, reported higher rates of respiratory system involvement [50,51]. It is encouraging that cardiovascular and neurological manifestations were least frequent, as the presence of these symptoms often corresponds with potentially life-threatening reactions [53].

The onset of symptoms in our study varied from an immediate reaction to 230 min after the start of the OFC, with an average onset time of 94.1 min, underscoring the importance of close and careful monitoring under supervision from the very beginning of the challenge.

Severity assessment in our study was based on H.A. Sampson’s grading system for food-induced anaphylaxis [25], with more than 90% of patients developing grade 1 or grade 2 symptoms during a failed OFC. The predominance of mild to moderate reactions was also recognized by Itazawa et al. [43], as well as Yanagida et al. [54]. Our findings may be partly justified by the prompt discontinuation of OFC and the timely administration of medications. For most positive OFCs, patients received antihistamine drugs and did not require any further treatment. Severe multisystemic reactions were recorded in less than 2% of all challenges, indicating that an OFC remains a safe procedure. It is possible that incremental dosing of the allergenic food enabled the prevention of more severe reaction occurrences, since challenges were stopped at the first signs of clinical reactivity. This resulted in a reduced total dose of the offending product, facilitated early intervention, and likely prevented progression to more serious reactions for many failed challenges.

Intramuscular epinephrine was administered to four children (6.1% of failed OFCs). The mean percentage of epinephrine administration in other studies ranged from 3% to 38% [33,35,43,44,52]. In our cohort, the patient who required two doses of intramuscular epinephrine in combination with nebulized epinephrine developed an acute respiratory tract infection on the day following the challenge. In clinical settings, infections as cofactors are commonly suspected to modulate and increase the severity of food-induced anaphylaxis occurrence [22,55,56], as well as reduce the threshold of an allergic reaction [57]. This emphasizes the importance of greater awareness when qualifying a patient for a procedure.

In our study, asthma was identified as a risk factor for OFC failure, similarly to Abrams et al. [19] and Ballini et al. [33]. Moreover, Chinthrajah et al. found the presence of exercise-induced asthma to contribute to the predictive ability of the severity of reaction to peanut OFCs [58]. Other authors discovered similar correlations between the history of asthma and occurrence of more severe reactions, including anaphylaxis during OFCs [35,48,59]. In a study by Aquilante et al., asthma was considered a risk factor for anaphylaxis in OFC, regardless of the allergen tested [50]. Bronchial asthma has consistently been reported as a risk factor for severe anaphylactic reactions, as well as fatal anaphylaxis, and may therefore contribute to an increased severity of symptoms during food challenges [60,61]. Although some authors did not discover a higher prevalence of asthma among children who failed an OFC than in those who passed the challenge [62,63], a strong link between asthma and food allergies has been widely observed [64] and such patients should be considered more likely to develop severe symptoms during an OFC.

Another risk factor associated with OFC failure discovered in our cohort was multi-food allergy, which is in accordance with findings by Sindher et al. [20]. Physicians should keep this in mind, since multiple food allergies are present in a substantial portion of the population of patients undergoing OFC procedures [32,65].

Our approach to stratify the OFC outcome based on the child’s age was motivated by evidence suggesting that age may influence the likelihood of a positive challenge outcome, particularly in cow’s milk and hen’s egg allergy, where tolerance often develops over time [66]. We did not observe a correlation between the patient’s age and challenge outcome, in line with findings from Ünsal et al. [35] and Koutlas et al. [44]. However, there is considerable discrepancy in the literature regarding this topic. In most cases, increasing age has been associated with a possibility of an OFC failure or more severe reactions during the procedure [19,33,46,48,62]. In contrast, Ogata et al. have found OFC-positive children to be younger than OFC-negative patients undergoing challenges with hen’s egg [67]. Differences between patients’ age in various study populations may account for this divergence. In addition, we discovered that all patients who refused to ingest the allergenic food during the challenge were under 3 years of age, underscoring the fact that OFC may be difficult to conduct in the youngest children, as infants experience periods of food refusal, even if they are in good health [68]. Our results suggest that, within our population, age alone may not be a reliable predictor of tolerance acquisition and should not be used in isolation to guide the decision to perform an oral food challenge.

Although atopic dermatitis is strongly associated with food allergies [69], and may be considered a risk factor for a positive OFC outcome [19,20,36], we did not find such associations, similarly to Yanagida et al. [52] and Kim et al. [46]. In the literature, the strongest association between atopic dermatitis and OFC failure is reported in children allergic to peanuts and tree nuts [36]. In our study population, the number of OFCs to peanuts and tree nuts was small, which may explain the lack of such correlation.

In contrast to other studies [36,46], neither a previous history of anaphylaxis to challenged food, nor to any food was found to be a risk factor for an OFC failure in our cohort. However, we discovered that a coexistence of asthma and previous history of anaphylaxis to any food was associated with the possibility of OFC failure. A similar correlation was found in patients with multi-food allergy and a history of anaphylaxis to any food and OFC failure. This underscores that, while a prior anaphylactic reaction alone may not significantly increase the risk of OFC failure, its coexistence with an atopic disease may contribute to a positive challenge outcome. In a study by Aquilante et al., prior anaphylaxis was not a risk factor for anaphylaxis during OFC [50]. Koutlas et al. did not find a correlation between a history of anaphylaxis to the challenged food and positive OFC either; however, such a correlation was discovered regarding the history of any anaphylaxis [44]. As mentioned above, a history of anaphylaxis shows divergent results in the literature, and therefore this aspect needs further investigation.

It is common knowledge that a positive family history of atopy increases the risk for atopic disease in offspring [70,71]; therefore, we intended to explore associations between this factor and OFC failure. However, such a correlation was not found.

As the majority of OFCs in our study were performed with cow’s milk and hen’s egg white, with baked forms, we sought to evaluate additional factors facilitating outcome prediction for challenges with these two foods. It has been widely reported in the literature that processing food influences its allergenicity, as most milk- and egg-allergic children are tolerant to the baked form [72,73]. The reduced allergenicity appears to be related to protein denaturation at elevated temperatures. Gantulga et al. identified the extensively decreased allergenicity of long-baked HEWP compared with boiled HEWP, as well as raw HEWP [74]. Moreover, a tolerance of baked milk and baked egg facilitates the development of tolerance and suggests that there will soon be a resolution of allergy to the unbaked form [75,76,77,78]. The incorporation of baked foods into a child’s diet is therefore valuable and considered a form of oral immunotherapy, with increasing amounts being ingested, followed by a subsequent switch to less processed forms [2,72,79,80,81].

It is well established that elevated sIgE levels are associated with an increased likelihood of an OFC failure. Several studies have demonstrated statistically significant differences in sIgE levels to food allergen extracts between patients who passed and those who did not pass OFCs or experienced severe symptoms during the procedure [62,67,82,83,84,85]. In part of our cohort, for whom serologic markers were available, a significant difference in sIgE levels was observed for HEWP in children undergoing challenges with baked HEWP. However, no such associations were identified for non-baked HEWP or for any form of CMP. Although sIgE levels mostly tended to be higher among patients who failed the challenge, the differences were not statistically significant. This may be due to the high heterogeneity of our study population and a relatively low sample size. Therefore, further research is needed to reassess this aspect. However, some authors have not discovered such differences either [19,51], underscoring that sIgE measurement has poor diagnostic efficacy, can have a high rate of false positives, and poorly predicts the severity of allergic reactions. This disparity is due in part to the poor sensitivity and specificity of the current testing available, as well as substantial differences in user interpretation [15]. Sasaki et al. discovered that in patients undergoing challenges with sIgE levels lower than 100 kU/L, positive OFCs showed significantly higher sIgE values for extracts of egg white protein than negative OFCs. However, there was no significant difference between positive and negative OFCs in the group with sIgE values ≥ 100 kU/L [86]. It is vital to note that severe reactions are possible even at low sIgE values to allergen extract. Gawryjołek et al. reported a case of a patient with low levels of HEWP-sIgE (0.75 kU/L), who developed severe symptoms of an allergic reaction during an OFC with a boiled hen’s egg [87]. In contrast, Greiwe described a case of a 3-year-old boy with a history of multiple food allergies with CMP-sIgE levels above 100 kU/L and HEWP-sIgE levels of 75.3 kU/L. Instead of condemning this patient to years of a strict elimination diet and social isolation, he was qualified for OFCs with baked milk and baked egg, followed by uncooked hen’s egg. The patient did not experience any symptoms of an allergic reaction during the procedures [15]. Findings from these studies underline that due to its poor diagnostic efficacy, sIgE serum testing may contribute to misdiagnosis, as well as an unnecessary elimination diet in children suspected of having a FA. Given the variability in sIgE levels, the same concentration may be sufficient to trigger anaphylaxis in one patient, while another with comparable sIgE levels may remain asymptomatic. Therefore, when determining eligibility for an OFC, it is advisable to consider not only the current sIgE levels but also the clinical presentation during the initial real-world anaphylactic episode.

In our cohort, the median concentrations of sIgE for CMP were significantly higher than those to HEWP among children who failed baked food challenges. This correlation was not observed in the unbaked allergen groups, likely due to the limited sample size. Concurrently, the mean cumulative reactive dose for baked CMP was more than twice as low as for HEWP. A similar trend was noted in the unbaked allergen group, with a *p* value approaching statistical significance, suggesting that this association may reach significance in studies with larger sample sizes. This is in accordance with other research findings, where eliciting doses were lower in challenges with CMP than HEWP [88,89]. Although an OFC is a planned and to some extent artificial procedure, our findings corroborate with results from Cichocka-Jarosz et al. [61], where similar reactive doses triggered real-life anaphylaxis to cow’s milk and hen’s egg. These findings indicate that CMP may exhibit greater allergenicity compared to HEWP, as smaller quantities of cow’s milk appear sufficient to elicit allergic reactions, which is consistent with observations reported in the aforementioned studies. Moreover, patients with baked milk tolerance may expose a greater risk of severe reactions than those who are tolerant to baked egg [89]. CMP is the most frequent food allergen in children under the age of 3 years [90], as well as the most common cause of anaphylaxis in this group. The propensity for this allergen to provoke lower respiratory reactions, including respiratory failure has been observed [65,91].

Although sIgE measurement is not a fully reliable predictive marker, as mentioned before, physicians need a tool which could help them predict the outcome of OFC and properly qualify or dispense a patient from the procedure. Several authors of the most recent studies have proposed cutoff sIgE levels for cow’s milk and hen’s egg challenges, with values ranging from 0.3 kU/L to 22.8 kU/L for CMP [20,44,82,90,92] and from 0.94 kU/L to 10.7 kU/L for HEWP [20,44,82,92]. There is no complete agreement between the numerous cutoffs defined by the various research findings [93]. As these thresholds were predominantly set for procedures with unbaked foods, we intended to develop a tool for a better prediction of outcomes for challenges with baked cow’s milk and hen’s egg. A cutoff sIgE level of 58.1 kU/L was set for baked milk challenges and a threshold of 11.3 kU/L for baked egg challenges. The cutoff levels were selected to maintain the highest specificity and consequently, a strong safety profile of the procedure. This, and a relatively small sample size, might have contributed to higher thresholds than previously reported in the literature. Moreover, in numerous studies, separate cutoff points were established for children in different age groups. In contrast, patients in our cohort were not stratified by age for this purpose. Our cohort also represents a highly allergic population with higher sIgE values than in children typically encountered in an average clinical setting. Finally, the fact that the OFCs were conducted with baked foods may have contributed to higher sIgE cutoff levels, as children undergoing procedures with baked cow’s milk and baked hen’s egg are generally more sensitized and have higher sIgE levels than patients challenged with unheated allergens [94]. It is difficult to establish reliable cutoff levels, since they are dependent on the disease prevalence and vary between populations. Therefore, our findings should be interpreted with caution. It is important to acknowledge that patients undergoing OFCs are frequently on highly restrictive diets due to prior, mostly severe, allergic reactions, particularly those undergoing their first baked food challenge. These results should not be considered predictive markers of tolerance development to food allergens, but rather as a supportive tool to aid clinicians in making informed decisions aimed at ensuring a safer OFC process for individual patients.

Our study had certain limitations. First, this study involved the use of an open OFC rather than a double-blind, placebo-controlled food challenge, which is the gold standard. However, open OFCs are acceptable for young children and for clinical practice. We did not observe too many subjective symptoms. Second, the number of analyzed OFCs was rather small. Pediatric OFCs are time-consuming and resource-intensive, compounded by a wide variety of allergens and common natural tolerance development. Consequently, many cases are diagnosed and resolved without OFCs, leading to a limited sample size. Third, this was a retrospective single-center study and relied on preexisting data. Oral food challenges from 2014 onward were included, at a time component-resolved diagnostics for food allergy (specific IgE to casein and ovomucoid) were not yet routinely performed and were available only for a subset of children. Therefore, to determine the optimal IgE cutoff values for predicting the outcome of oral food challenges, specific IgE to cow’s milk and hen’s egg white extracts was used in the analysis. Although in our study we did not perform measurements of specific IgE to casein and ovomucoid, it is important to note that, according to the most recent systematic review and meta-analysis by Riggioni et al. [12], specific IgE to food extracts demonstrates both high sensitivity and good specificity in the diagnosis of food allergy. In particular, sIgE to cow’s milk extract shows higher sensitivity while maintaining acceptable specificity, and IgE to egg white extract has been shown to have comparable or even better diagnostic accuracy than sIgE to ovomucoid. These findings support the clinical utility of extract-based testing in predicting the outcomes of oral food challenges.

## 5. Conclusions

In this study, we intended to share our experiences in providing oral food challenges in a pediatric population. Our findings confirm that OFCs remain a critical tool in the diagnosis and management of food allergies, with cow’s milk and hen’s egg being the most implicated allergens in failed challenges. Importantly, we identified asthma and multiple food allergies as significant risk factors for OFC failure. These results underscore the need for heightened caution and individualized risk assessment when considering OFCs in children with these conditions. Enhancing pre-challenge evaluation protocols, we included a detailed medical history and specific IgE levels. Incorporating these considerations into clinical practice may improve patient safety and optimize diagnostic accuracy. Our further prospective observations will focus on refining predictive tools and identifying biomarkers that can stratify risk in children undergoing OFCs.

## Figures and Tables

**Table 1:** Characteristics of the study population.

Feature	Value
**Number of patients**	205 *
**Mean age, SD; age range (years)**	5.7 ± 3.1; 1.2–17.8
**Gender, n (%)**	
Male	135 (65.9%)
Female	70 (34.1%)
**Allergen challenged in the OFC, n (%)**	
Cow’s milk protein	103 (50.2%)
Hen’s egg white protein	84 (41.0%)
Peanuts and tree nuts (hazelnut, cashew, and walnut)	7 (3.4%)
Peanuts	3 (1.5%)
Hazelnuts	2 (1.0%)
Cashew	1 (0.5%)
Walnut	1 (0.5%)
Gluten	3 (1.5%)
Hen’s egg yolk	4 (2.0%)
Other (chicken, cod, banana, and potato)	4 (2.0%)
**Mean time from food consumption to reaction occurrence, SD, range (minutes)**	94.1 ± 50.4; 0–230
**Accompanying atopic diseases**	
Multi-food allergy	152 (74.1%)
Atopic dermatitis	124 (60.5%)
Asthma	101 (49.3%)
Family history of atopy	85 (41.5%)
Previous history of anaphylaxis to any food	158 (77.1%)
Previous history of anaphylaxis to challenged food	110 (53.7%)

* Including five patients with inconclusive OFC results. SD—standard deviation; OFC—oral food challenge.

**Table 2:** Characteristics of CMP and HEWP groups.

Feature	CMP (n = 103) *	HEWP (n = 84) *	*p*
**Mean age, SD; age range (years)**	5.41	5.54	0.52
Min	1.18	1.54	
Max	17.84	16.54	
SD	3.03	2.91	
**Gender, n (%)**			
Male	70 (67.96%)	55 (65.48%)	0.72
Female	33 (32.04%)	29 (34.52%)	
**Mean time from consumption to reaction occurrence, SD, range (minutes)**	97.28	95.86	0.88
Min	0	0	
Max	230	180	
SD	56.3	43.96	
**Accompanying atopic diseases**			
Multi-food allergy	74 (71.84%)	66 (78.57%)	0.29
Atopic dermatitis	55(53.4%)	58 (69.05%)	**0.03**
Asthma	54 (52.43%)	38 (45.24%)	0.33
Family history of atopy	44 (42.72%)	36 (42.86%)	0.99
Previous history of anaphylaxis to any food	79 (76.7%)	64 (79.19%)	0.94
Previous history of anaphylaxis to challenged food	63 (61.17%)	40 (47.62%)	0.06

* Including patients with inconclusive results. CMP—cow’s milk protein; HEWP—hen’s egg white protein; SD—standard deviation; min—minimum; and max—maximum. Statistically significant *p* values (*p* < 0.05) are in bold.

**Table 3:** Outcomes of all oral food challenges by allergen and its preparation form.

Allergen Challenged	Total No. of OFCs *	No. of Failed OFCs (%)
**Milk:**	103	32 (31.1%)
Baked milk	66	20 (30.3%)
Non-baked milk:	37	12 (32.4%)
Fermented milk	14	5 (35.7%)
Plain fresh milk	7	1 (14.3%)
Modified infant formula	7	3 (42.9%)
Heated fresh milk	6	1 (16.7%)
Other forms	3	2 (66.7%)
**Hen’s Egg White:**	84	29 (34.5%)
Baked	60	21 (35.0%)
Cooked	24	8 (33.3%)
**Hen’s Egg Yolk:**	4	1 (25.0%)
Cooked	3	1 (33.3%)
Baked	1	0 (0.0%)
**Peanuts and tree nuts:**	7	1 (14.3%)
Peanuts	3	1 (33.3%)
Hazelnuts	2	0 (0.0%)
Cashew	1	0 (0.0%)
Walnut	1	0 (0.0%)
**Gluten**	3	2 (66.7%)
**Other:**	4	1 (25.0%)
Chicken	1	1 (100.0%)
Cod	1	0 (0.0%)
Banana	1	0 (0.0%)
Potato	1	0 (0.0%)

* Including patients with inconclusive results. OFC—oral food challenge.

**Table 4:** Cumulative reactive dose expressed in grams of allergenic protein in failed CMP and HEWP OFCs.

	N	Cow’s Milk Protein [g]	N	Hen’s Egg White Protein [g]	*p* Value
**Baked**	20	0.27 [0.135–0.54]	21	0.58 [0.29–1.17]	**0.01**
**Non-baked**	12	1.16 [0.12–1.66]	8	2.68 [0.765–3.83]	0.06

Data are presented as median and [interquartile range]. N—sample size in each group. CMP—cow’s milk protein; HEWP—hen’s egg white protein. Statistically significant *p* values (*p* < 0.05) are in bold.

**Table 5:** Influence of various factors on the OFC outcome by mean logistic regressions (N = 200).

Variables	Coef.	Std. Err.	z	*p* > |z|	[0.025	0.975]
**Asthma**	0.67	0.31	2.19	**0.03**	0.07	1.27
**Atopic dermatitis**	0.13	0.31	0.43	0.67	−0.47	0.74
**Previous history of anaphylaxis** **to any food**	0.61	0.36	1.69	0.09	−0.10	1.31
**Previous history of anaphylaxis to challenged food**	0.49	0.29	1.66	0.10	−0.09	1.07
**Multi-food allergy**	0.90	0.39	2.31	**0.02**	0.14	1.67

**Coef.**—The estimated coefficient of the independent variable in the ordinal logistic regression model. It represents the effect size of the predictor on the dependent variable. **Std. Err.**—The standard error of the coefficient estimate, indicating the level of variability in the estimate. A smaller value suggests higher precision. **z**—The z-score (test statistic), which measures how many standard deviations the coefficient is away from zero. Higher absolute values indicate stronger relationships. ***p* > |z|**—The *p* value, representing the probability that the coefficient is different from zero due to random chance. A value below 0.05 typically suggests statistical significance. **0.025**—The lower bound of the 95% confidence interval for the coefficient estimate. **0.975**—The upper bound of the 95% confidence interval for the coefficient estimate. Statistically significant *p* values (*p* < 0.05) are in bold.

**Table 6:** Specific IgE levels for passed and failed OFCs in baked and non-baked allergen groups.

	Baked					Non-Baked				
	N	Passed	N	Failed	*p* Value	N	Passed	N	Failed	*p* Value
**CMP-specific IgE [kU/L]**	34	18.80 [5.69–35.90]	17	45.00 [6.77–65.90]	0.20	19	1.53 [0.25–4.05]	8	1.28 [0.39–5.88]	0.83
**HEWP-specific IgE [kU/L]**	28	6.48 [3.28–18.90]	13	18.90 [5.87–35.40]	**0.01**	12	1.12 [0.53–3.45]	7	23.90 [3.22–39.00]	0.12

Data are presented as median and [interquartile range]. N—sample size in each group. CMP—cow’s milk protein; HEWP—hen’s egg white protein. Statistically significant *p* values (*p* < 0.05) are in bold.

**Table 7:** CMP- and HEWP-specific IgE levels in failed baked and non-baked OFCs.

	N	CMP-Specific IgE [kU/L]	N	HEWP-Specific IgE [kU/L]	*p* Value
**Baked**	17	58.1 [19.4–100.0]	13	18.9 [5.87–35.4]	**0.02**
**Non-baked**	8	3.53 [1.13–8.58]	7	23.9 [3.22–39.0]	0.06

Data are presented as median and [interquartile range]. N—sample size in each group. CMP—cow’s milk protein; HEWP—hen’s egg white protein; and OFC—oral food challenge. Statistically significant *p* values (*p* < 0.05) are in bold.

## Data Availability

Data are not openly available due to ethical/privacy restrictions as permission for data sharing was not provided by the participants or the Ethical Committee.

## Abbreviations

The following abbreviations are used in this manuscript:

FA	food allergy
OFC	oral food challenge
CMP	cow’s milk protein
HEWP	hen’s egg white protein
ROC	receiver operating characteristic
DBPCFC	double-blind, placebo-controlled food challenge
sIgE	specific IgE
AUC	area under curve
CRD	component-resolved diagnostics

## References

[1] Muraro A., de Silva D., Halken S., Worm M., Khaleva E., Arasi S., Dunn-Galvin A., Nwaru B.I., De Jong N.W., Rodríguez Del Río P. (2022). Managing Food Allergy: GA2LEN Guideline 2022. World Allergy Organ. J..

[2] Barni S., Liccioli G., Sarti L., Giovannini M., Novembre E., Mori F. (2020). Immunoglobulin E (IgE)-Mediated Food Allergy in Children: Epidemiology, Pathogenesis, Diagnosis, Prevention, and Management. Medicina.

[3] Sampath V., Abrams E.M., Adlou B., Akdis C., Akdis M., Brough H.A., Chan S., Chatchatee P., Chinthrajah R.S., Cocco R.R. (2021). Food Allergy across the Globe. J. Allergy Clin. Immunol..

[4] Sicherer S.H., Sampson H.A. (2018). Food Allergy: A Review and Update on Epidemiology, Pathogenesis, Diagnosis, Prevention, and Management. J. Allergy Clin. Immunol..

[5] Botha M., Basera W., Facey-Thomas H.E., Gaunt B., Gray C.L., Ramjith J., Watkins A., Levin M.E. (2019). Rural and Urban Food Allergy Prevalence from the South African Food Allergy (SAFFA) Study. J. Allergy Clin. Immunol..

[6] Sigurdardottir S.T., Jonasson K., Clausen M., Lilja Bjornsdottir K., Sigurdardottir S.E., Roberts G., Grimshaw K., Papadopoulos N.G., Xepapadaki P., Fiandor A. (2021). Prevalence and Early-Life Risk Factors of School-Age Allergic Multimorbidity: The EuroPrevall-IFAAM Birth Cohort. Allergy Eur. J. Allergy Clin. Immunol..

[7] Grabenhenrich L., Trendelenburg V., Bellach J., Yürek S., Reich A., Fiandor A., Rivero D., Sigurdardottir S., Clausen M., Papadopoulos N.G. (2020). Frequency of Food Allergy in School-Aged Children in Eight European Countries—The EuroPrevall-IFAAM Birth Cohort. Allergy Eur. J. Allergy Clin. Immunol..

[8] Spolidoro G.C.I., Amera Y.T., Ali M.M., Nyassi S., Lisik D., Ioannidou A., Rovner G., Khaleva E., Venter C., van Ree R. (2023). Frequency of Food Allergy in Europe: An Updated Systematic Review and Meta-Analysis. Allergy Eur. J. Allergy Clin. Immunol..

[9] Knyziak-Mędrzycka I., Majsiak E., Gromek W., Kozłowska D., Swadźba J., Beata B.J., Kurzawa R., Cukrowska B. (2024). The Sensitization Profile for Selected Food Allergens in Polish Children Assessed with the Use of a Precision Allergy Molecular Diagnostic Technique. Int. J. Mol. Sci..

[10] Lyons S.A., Clausen M., Knulst A.C., Ballmer-Weber B.K., Fernandez-Rivas M., Barreales L., Bieli C., Dubakiene R., Fernandez-Perez C., Jedrzejczak-Czechowicz M. (2020). Prevalence of Food Sensitization and Food Allergy in Children Across Europe. J. Allergy Clin. Immunol. Pract..

[11] Krzych-Fałta E., Białek S., Sybilski A.J., Tylewicz A., Samoliński B., Wojas O. (2023). Differential Diagnostics of Food Allergy as Based on Provocation Tests and Laboratory Diagnostic Assays. Postep. Dermatol. Alergol..

[12] Riggioni C., Ricci C., Moya B., Wong D., van Goor E., Bartha I., Buyuktiryaki B., Giovannini M., Jayasinghe S., Jaumdally H. (2024). Systematic Review and Meta-Analyses on the Accuracy of Diagnostic Tests for IgE-Mediated Food Allergy. Allergy Eur. J. Allergy Clin. Immunol..

[13] Calvani M., Bianchi A., Reginelli C., Peresso M., Testa A. (2019). Oral Food Challenge. Medicina.

[14] Hsu E., Soller L., Abrams E.M., Protudjer J.L.P., Mill C., Chan E.S. (2020). Oral Food Challenge Implementation: The First Mixed-Methods Study Exploring Barriers and Solutions. J. Allergy Clin. Immunol. Pract..

[15] Greiwe J. (2019). Oral Food Challenges in Infants and Toddlers. Immunol. Allergy Clin. N. Am..

[16] Santos A.F., Riggioni C., Agache I., Akdis C.A., Akdis M., Alvarez-Perea A., Alvaro-Lozano M., Ballmer-Weber B., Barni S., Beyer K. (2023). EAACI Guidelines on the Diagnosis of IgE-Mediated Food Allergy. Allergy Eur. J. Allergy Clin. Immunol..

[17] Eiwegger T., Hung L., San Diego K.E., O’Mahony L., Upton J. (2019). Recent Developments and Highlights in Food Allergy. Allergy Eur. J. Allergy Clin. Immunol..

[18] Yanagida N., Sato S., Asaumi T., Ogura K., Ebisawa M. (2017). Risk Factors for Severe Reactions during Double-Blind Placebo-Controlled Food Challenges. Int. Arch. Allergy Immunol..

[19] Abrams E.M., Becker A.B. (2017). Oral Food Challenge Outcomes in a Pediatric Tertiary Care Center. Allergy Asthma Clin. Immunol..

[20] Sindher S., Long A.J., Purington N., Chollet M., Slatkin S., Andorf S., Tupa D., Kumar D., Woch M.A., O’Laughlin K.L. (2018). Analysis of a Large Standardized Food Challenge Data Set to Determine Predictors of Positive Outcome across Multiple Allergens. Front. Immunol..

[21] Bird J.A., Leonard S., Groetch M., Assa’ad A., Cianferoni A., Clark A., Crain M., Fausnight T., Fleischer D., Green T. (2020). Conducting an Oral Food Challenge: An Update to the 2009 Adverse Reactions to Foods Committee Work Group Report. J. Allergy Clin. Immunol. Pract..

[22] Sampson H.A., Gerth Van Wijk R., Bindslev-Jensen C., Sicherer S., Teuber S.S., Burks A.W., Dubois A.E.J., Beyer K., Eigenmann P.A., Spergel J.M. (2012). Standardizing Double-Blind, Placebo-Controlled Oral Food Challenges: American Academy of Allergy, Asthma & Immunology-European Academy of Allergy and Clinical Immunology PRACTALL Consensus Report. J. Allergy Clin. Immunol..

[23] Nowak-Wegrzyn A., Assa’ad A.H., Bahna S.L., Bock S.A., Sicherer S.H., Teuber S.S. (2009). Work Group Report: Oral Food Challenge Testing. J. Allergy Clin. Immunol..

[24] Leonard S.A., Caubet J.C., Kim J.S., Groetch M., Nowak-Wegrzyn A. (2015). Baked Milk- and Egg-Containing Diet in the Management of Milk and Egg Allergy. J. Allergy Clin. Immunol. Pract..

[25] Sampson H.A. (2003). Anaphylaxis and Emergency Treatment. Pediatrics.

[26] Dölle-Bierke S., Höfer V., Francuzik W., Näher A.F., Bilo M.B., Cichocka-Jarosz E., Lopes de Oliveira L.C., Fernandez-Rivas M., García B.E., Hartmann K. (2023). Food-Induced Anaphylaxis: Data from the European Anaphylaxis Registry. J. Allergy Clin. Immunol. Pract..

[27] de Almeida Kotchetkoff E.C., de Oliveira L.C.L., Sarni R.O.S. (2024). Elimination Diet in Food Allergy: Friend or Foe?. J. Pediatr. (Rio J.).

[28] Şengül Emeksiz Z., Ertuğrul A., Uygun S.D., Özmen S. (2023). Evaluation of Emotional, Behavioral, and Clinical Characteristics of Children Aged 1–5 with a History of Food-Related Anaphylaxis. Pediatr. Neonatol..

[29] Assa’ad A.H. (2020). Oral Food Challenges. J. Food Allergy.

[30] Murai H., Irahara M., Sugimoto M., Takaoka Y., Takahashi K., Wada T., Yamamoto-Hanada K., Okafuji I., Yamada Y., Futamura M. (2022). Is Oral Food Challenge Useful to Avoid Complete Elimination in Japanese Patients Diagnosed with or Suspected of Having IgE-Dependent Hen’s Egg Allergy? A Systematic Review. Allergol. Int..

[31] Maeda M., Kuwabara Y., Tanaka Y., Nishikido T., Hiraguchi Y., Yamamoto-Hanada K., Okafuji I., Yamada Y., Futamura M., Ebisawa M. (2022). Is Oral Food Challenge Test Useful for Avoiding Complete Elimination of Cow’s Milk in Japanese Patients with or Suspected of Having IgE-Dependent Cow’s Milk Allergy?. Allergol. Int..

[32] Correa N., Protudjer J.L.P., Hsu E., Soller L., Chan E.S., Kim H., Jeimy S. (2022). Canadian Parent Perceptions of Oral Food Challenges: A Qualitative Analysis. Pediatr. Allergy Immunol..

[33] Ballini G., Gavagni C., Guidotti C., Ciolini G., Liccioli G., Giovannini M., Sarti L., Ciofi D., Novembre E., Mori F. (2021). Frequency of Positive Oral Food Challenges and Their Outcomes in the Allergy Unit of a Tertiary-Care Pediatric Hospital. Allergol. Immunopathol..

[34] Ogata M., Kido J., Watanabe S., Yoshida T., Nishi N., Shimomura S., Hirai N., Tanaka K., Mizukami T., Yanai M. (2024). The Efficacy and Safety of Stepwise Oral Food Challenge in Children with Cow’s Milk Allergy. Int. Arch. Allergy Immunol..

[35] Ünsal H., Gökçe Ö.B.G., Ocak M., Akarsu A., Şahiner Ü.M., Soyer Ö., Şekerel B.E. (2021). Oral Food Challenge in IgE Mediated Food Allergy in Eastern Mediterranean Children. Allergol. Immunopathol..

[36] Jacob J.G., Fernando S.L., Nickolls C., Li J. (2023). Oral Food Challenge Outcomes in Children and Adolescents in a Tertiary Centre: A 5-Year Experience. J. Paediatr. Child. Health.

[37] Emeksiz Z.S., Ertugrul A., Ozmen S., Cavkaytar O., Ercan N., Bostancl I.B. (2021). Is Oral Food Challenge as Safe Enough as It Seems?. J. Trop. Pediatr..

[38] Leffler J., Stumbles P.A., Strickland D.H. (2018). Immunological Processes Driving IgE Sensitisation and Disease Development in Males and Females. Int. J. Mol. Sci..

[39] Uekert S.J., Akan G., Evans M.D., Li Z., Roberg K., Tisler C., DaSilva D., Anderson E., Gangnon R., Allen D.B. (2006). Sex-Related Differences in Immune Development and the Expression of Atopy in Early Childhood. J. Allergy Clin. Immunol..

[40] Su Y., Wen J., Zhang H., Zou Z., Cai Y., Zhang C. (2023). Clinical Characteristics of Anaphylaxis in Children Aged 0–16 Years in Xi’an, China. Int. Arch. Allergy Immunol..

[41] Gaspar Â., Santos N., Faria E., Pereira A.M., Gomes E., Câmara R., Rodrigues-Alves R., Borrego L.M., Carrapatoso I., Carneiro-Leão L. (2021). Anaphylaxis in Children and Adolescents: The Portuguese Anaphylaxis Registry. Pediatr. Allergy Immunol..

[42] Honda A., Imai T., Okada Y., Maeda M., Kamiya T. (2024). Severe Anaphylaxis Requiring Continuous Adrenaline Infusion during Oral Food Challenge: A Case Series. Ann. Allergy Asthma Immunol..

[43] Itazawa T., Adachi Y., Takahashi Y., Miura K., Uehara Y., Kameda M., Kitamura T., Kuzume K., Tezuka J., Ito K. (2020). The Severity of Reaction after Food Challenges Depends on the Indication: A Prospective Multicenter Study. Pediatr. Allergy Immunol..

[44] Koutlas N., Stallings A., Hall G., Zhou C., Kim-Chang J., Mousallem T. (2024). Pediatric Oral Food Challenges in the Outpatient Setting: A Single-Center Experience. J. Allergy Clin. Immunol. Glob..

[45] Yagmur I.T., Celik I.K., Topal O.Y., Toyran M., Civelek E., Misirlioglu E.D. (2023). Development of Anaphylaxis upon Oral Food Challenge and Drug Provocation Tests in Pediatric Patients. Allergy Asthma Proc..

[46] Kim H., Jeong K., Park M., Roh Y.Y., Jung J.H., Kim S.Y., Kim J.D., Kim M.J., Kim Y.H., Sohn M.H. (2024). Predicting the Outcome of Pediatric Oral Food Challenges for Determining Tolerance Development. Allergy Asthma Immunol. Res..

[47] Pouessel G., Jean-Bart C., Deschildre A., Van der Brempt X., Tanno L.K., Beaumont P., Dumond P., Sabouraud-Leclerc D., Beaudouin E., Ramdane N. (2020). Food-Induced Anaphylaxis in Infancy Compared to Preschool Age: A Retrospective Analysis. Clin. Exp. Allergy.

[48] Kennedy K., Alfaro M.K.C., Spergel Z.C., Dorris S.L., Spergel J.M., Capucilli P. (2021). Differences in Oral Food Challenge Reaction Severity Based on Increasing Age in a Pediatric Population. Ann. Allergy Asthma Immunol..

[49] Capucilli P., Cianferoni A., Fiedler J., Gober L., Pawlowski N., Ram G., Saltzman R., Spergel J.M., Heimall J. (2018). Differences in Egg and Milk Food Challenge Outcomes Based on Tolerance to the Baked Form. Ann. Allergy Asthma Immunol..

[50] Aquilante B.P., Castro A.P.B.M., Yonamine G.H., de Barros Dorna M., Barp M.F., Martins T.P.d.R., Pastorino A.C. (2023). IgE-Mediated Cow’s Milk Allergy in Brazilian Children: Outcomes of Oral Food Challenge. World Allergy Organ. J..

[51] Esteban C.A., Shreffler W.G., Virkud Y.V., Pistiner M. (2020). Oral Food Challenge Outcomes in Children under 3 Years of Age. J. Allergy Clin. Immunol. Pract..

[52] Yanagida N., Sato S., Nagakura K., Takahashi K., Fusayasu N., Miura Y., Itonaga T., Ogura K., Ebisawa M. (2023). Relationship between Serum Allergen-Specific Immunoglobulin E and Threshold Dose in an Oral Food Challenge. Pediatr. Allergy Immunol..

[53] Triggiani M., Patella V., Staiano R.I., Granata F., Marone G. (2008). Allergy and the Cardiovascular System. Clin. Exp. Immunol..

[54] Yanagida N., Minoura T., Sato S., Takahashi K., Nagakura K., Ogura K., Itonaga T., Miura Y., Fusayasu N., Ebisawa M. (2024). Timing of Initial Symptom Onset during Milk and Wheat Challenges: A Retrospective Study. Immun. Inflamm. Dis..

[55] Shin M. (2021). Food Allergies and Food-Induced Anaphylaxis: Role of Cofactors. Clin. Exp. Pediatr..

[56] Benedé S., Garrido-Arandia M., Martín-Pedraza L., Bueno C., Díaz-Perales A., Villalba M. (2017). Multifactorial Modulation of Food-Induced Anaphylaxis. Front. Immunol..

[57] Upton J.E.M., Bird J.A. (2020). Oral Food Challenges: Special Considerations. Ann. Allergy Asthma Immunol..

[58] Chinthrajah R.S., Purington N., Andorf S., Rosa J.S., Mukai K., Hamilton R., Smith B.M., Gupta R., Galli S.J., Desai M. (2018). Development of a Tool Predicting Severity of Allergic Reaction during Peanut Challenge. Ann. Allergy Asthma Immunol..

[59] Purington N., Chinthrajah R.S., Long A., Sindher S., Andorf S., O’Laughlin K., Woch M.A., Scheiber A., Assa’Ad A., Pongracic J. (2018). Eliciting Dose and Safety Outcomes from a Large Dataset of Standardized Multiple Food Challenges. Front. Immunol..

[60] Olabarri M., Vazquez P., Gonzalez-Posada A., Sanz N., Gonzalez-Peris S., Diez N., Vinuesa A., Martinez-Indart L., Benito J., Mintegi S. (2020). Risk Factors for Severe Anaphylaxis in Children. J. Pediatr..

[61] Cichocka-Jarosz E., Dölle-Bierke S., Jedynak-Wąsowicz U., Sabouraud-Leclerc D., Köhli A., Lange L., Papadopoulos N.G., Hourihane J., Nemat K., Scherer Hofmeier K. (2023). Cow’s Milk and Hen’s Egg Anaphylaxis: A Comprehensive Data Analysis from the European Anaphylaxis Registry. Clin. Transl. Allergy.

[62] Nishino M., Yanagida N., Sato S., Nagakura K., Takahashi K., Ogura K., Ebisawa M. (2022). Risk Factors for Failing a Repeat Oral Food Challenge in Preschool Children with Hen’s Egg Allergy. Pediatr. Allergy Immunol..

[63] Taniuchi S., Sakai R., Nishida T., Goma M., Mitomori M., Imaide A., Enomoto M., Nishino M., Okizuka Y., Kido H. (2023). The Combination of Binding Avidity of Ovomucoid-Specific IgE Antibody and Specific IgG4 Antibody Can Predict Positive Outcomes of Oral Food Challenges during Stepwise Slow Oral Immunotherapy in Children with Hen’s Egg Allergy. Nutrients.

[64] Cunico D., Giannì G., Scavone S., Buono E.V., Caffarelli C. (2024). The Relationship Between Asthma and Food Allergies in Children. Children.

[65] Yonkof J.R., Mikhail I.J., Prince B.T., Stukus D. (2021). Delayed and Severe Reactions to Baked Egg and Baked Milk Challenges. J. Allergy Clin. Immunol. Pract..

[66] Savage J., Sicherer S., Wood R. (2016). The Natural History of Food Allergy. J. Allergy Clin. Immunol. Pract..

[67] Ogata M., Kido J., Yoshida T., Nishi N., Shimomura S., Hirai N., Mizukami T., Yanai M., Nakamura K. (2024). The Efficacy and Safety of Stepwise Oral Food Challenge in Children with Hen’s Egg Allergy. Allergy Asthma Clin. Immunol..

[68] Hill S.A., Nurmatov U., DunnGalvin A., Reese I., Vieira M.C., Rommel N., Dupont C., Venter C., Cianferoni A., Walsh J. (2024). Feeding Difficulties in Children with Food Allergies: An EAACI Task Force Report. Pediatr. Allergy Immunol..

[69] Domínguez O., Plaza A.M., Alvaro M. (2019). Relationship Between Atopic Dermatitis and Food Allergy. Curr. Pediatr. Rev..

[70] Mohammad Tariq S., Marie Matthews S., Abe Hakim E., Stevens M., Hasan Arshad S., Wallace Hide D. (1998). The Prevalence of and Risk Factors for Atopy in Early Childhood: A Whole Population Birth Cohort Study. J. Allergy Clin. Immunol..

[71] Manti S., Galletta F., Bencivenga C.L., Bettini I., Klain A., D’Addio E., Mori F., Licari A., Miraglia Del Giudice M., Indolfi C. (2024). Food Allergy Risk: A Comprehensive Review of Maternal Interventions for Food Allergy Prevention. Nutrients.

[72] Gonzalez P.M., Cassin A.M., Durban R., Upton J.E.M. (2025). Effects of Food Processing on Allergenicity. Curr. Allergy Asthma Rep..

[73] Nowak-Wegrzyn A., Bloom K.A., Sicherer S.H., Shreffler W.G., Noone S., Wanich N., Sampson H.A. (2008). Tolerance to Extensively Heated Milk in Children with Cow’s Milk Allergy. J. Allergy Clin. Immunol..

[74] Gantulga P., Lee J., Jeong K., Jeon S.A., Lee S. (2024). Variation in the Allergenicity of Scrambled, Boiled, Short-Baked and Long-Baked Egg White Proteins. J. Korean Med. Sci..

[75] Gruzelle V., Juchet A., Martin-Blondel A., Michelet M., Chabbert-Broue A., Didier A. (2020). Benefits of Baked Milk Oral Immunotherapy in French Children with Cow’s Milk Allergy. Pediatr. Allergy Immunol..

[76] Kim J.S., Nowak-Wgrzyn A., Sicherer S.H., Noone S., Moshier E.L., Sampson H.A. (2011). Dietary Baked Milk Accelerates the Resolution of Cow’s Milk Allergy in Children. J. Allergy Clin. Immunol..

[77] Giannetti A., Toschi Vespasiani G., Ricci G., Miniaci A., Di Palmo E., Pession A. (2021). Cow’s Milk Protein Allergy as a Model of Food Allergies. Nutrients.

[78] Leonard S.A., Sampson H.A., Sicherer S.H., Noone S., Moshier E.L., Godbold J., Nowak-Wegrzyn A. (2012). Dietary Baked Egg Accelerates Resolution of Egg Allergy in Children. J. Allergy Clin. Immunol..

[79] Upton J.E.M., Wong D., Nowak-Wegrzyn A. (2024). Baked Milk and Egg Diets Revisited. Ann. Allergy Asthma Immunol..

[80] Buyuktiryaki B., Soyer O., Bingol G., Can C., Nacaroglu H.T., Bingol A., Arik Yilmaz E., Aydogan M., Sackesen C. (2024). Milk Ladder: Who? When? How? Where? With the Lowest Risk of Reaction. Front. Allergy.

[81] Meyer R., Venter C., Bognanni A., Szajewska H., Shamir R., Nowak-Wegrzyn A., Fiocchi A., Vandenplas Y. (2023). World Allergy Organization (WAO) Diagnosis and Rationale for Action against Cow’s Milk Allergy (DRACMA) Guideline Update—VII—Milk Elimination and Reintroduction in the Diagnostic Process of Cow’s Milk Allergy. World Allergy Organ. J..

[82] De Boer R., Cartledge N., Lazenby S., Tobias A., Chan S., Fox A.T., Santos A.F. (2020). Specific IgE as the Best Predictor of the Outcome of Challenges to Baked Milk and Baked Egg. J. Allergy Clin. Immunol. Pract..

[83] Yanagida N., Sato S., Ebisawa M. (2023). Relationship between Eliciting Doses and the Severity of Allergic Reactions to Food. Curr. Opin. Allergy Clin. Immunol..

[84] Yanagida N., Sato S., Takahashi K., Nagakura K.I., Asaumi T., Ogura K., Ebisawa M. (2018). Increasing Specific Immunoglobulin E Levels Correlate with the Risk of Anaphylaxis during an Oral Food Challenge. Pediatr. Allergy Immunol..

[85] Coimbra M.R., Araújo L.M.L., Filho N.A.R. (2023). Oral Food Challenge in Children with Contact Urticaria in Reaction to Cow’s Milk. Allergol. Immunopathol..

[86] Sasaki Y., Matsunami K., Kondo M., Matsukuma E., Imamura A., Kaneko H. (2024). Oral Food Challenge Test Results of Patients with Food Allergy with Specific IgE Levels >100 UA/Ml. Biomed. Rep..

[87] Gawryjołek J., Wycech A., Smyk A., Krogulska A. (2021). Difficulties in Interpretation of Oral Food Challenge Results. Postep. Dermatol. Alergol..

[88] Valluzzi R.L., Riccardi C., Urbani S., Ursi D., Zavettieri D., Di Girolamo F., Dahdah L., Calandrelli V., Fierro V., Fiocchi A. (2025). The Baked Side: Cow’s Milk and Egg Protein Threshold Dose Distributions in Children Reacting to Baked Milk and Baked Egg. World Allergy Organ. J..

[89] Valluzzi R.L., Riccardi C., Arasi S., Piscitelli A.L., Calandrelli V., Dahdah L., Fierro V., Mennini M., Fiocchi A. (2022). Cow’s Milk and Egg Protein Threshold Dose Distributions in Children Tolerant to Beef, Baked Milk, and Baked Egg. Allergy Eur. J. Allergy Clin. Immunol..

[90] Tosca M.A., Schiavetti I., Olcese R., Trincianti C., Ciprandi G. (2023). Molecular Allergy Diagnostics in Children with Cow’s Milk Allergy: Prediction of Oral Food Challenge Response in Clinical Practice. J. Immunol. Res..

[91] Upton J., Alvaro M., Nadeau K. (2019). A Perspective on the Pediatric Death from Oral Food Challenge Reported from the Allergy Vigilance Network. Allergy Eur. J. Allergy Clin. Immunol..

[92] Sampson H.A., Arasi S., Bahnson H.T., Ballmer-Weber B., Beyer K., Bindslev-Jensen C., Bird J.A., Blumchen K., Davis C., Ebisawa M. (2024). AAAAI–EAACI PRACTALL: Standardizing Oral Food Challenges—2024 Update. Pediatr. Allergy Immunol..

[93] Anagnostou K. (2018). Safety of Oral Food Challenges in Early Life. Children.

[94] Sirin Kose S., Asilsoy S., Uzuner N., Karaman O., Anal O. (2019). Outcomes of Baked Milk and Egg Challenge in Cow’s Milk and Hen’s Egg Allergy: Can Tolerance Be Predicted with Allergen-Specific IgE and Prick-to-Prick Test?. Int. Arch. Allergy Immunol..

